# A mixed methods analysis evaluating an alcohol health champion community intervention: How do newly trained champions perceive and understand their training and role?

**DOI:** 10.1111/hsc.13717

**Published:** 2022-01-17

**Authors:** Suzy C. Hargreaves, Cathy Ure, Elizabeth J. Burns, Margaret Coffey, Suzanne Audrey, Kate Ardern, Penny A. Cook

**Affiliations:** ^1^ 7046 School of Health and Society University of Salford Salford UK; ^2^ 1980 Bristol Medical School University of Bristol Bristol UK; ^3^ 11622 Wigan Council Wigan UK

**Keywords:** alcohol, brief intervention, community, licensing, public health

## Abstract

Globally, alcohol harm is recognised as one of the greatest population risks and reducing alcohol harm is a key priority for the UK Government. The Communities in Charge of Alcohol (CICA) programme took an asset‐based approach in training community members across nine areas to become alcohol health champions (AHCs); trained in how to have informal conversations about alcohol and get involved with alcohol licensing. This paper reports on the experiences of AHCs taking part in the training through the analysis of: questionnaires completed pre‐ and post‐training (*n* = 93) and semi‐structured interviews with a purposive sample of five AHCs who had started their role. Questionnaires explored: characteristics of AHCs, perceived importance of community action around alcohol and health, and confidence in undertaking their role. Following training AHCs felt more confident to talk about alcohol harms, give brief advice and get involved in licensing decisions. Interviews explored: AHCs’ experiences of the training, barriers and facilitators to the adoption of their role, and how they made sense of their role. Four overarching themes were identified through thematic analysis taking a framework approach: (a) perceptions of AHC training; (b) applying knowledge and skills in the AHC role; (c) barriers and facilitators to undertaking the AHC role; and (d) sustaining the AHC role. Findings highlight the challenges in establishing AHC roles can be overcome by combining the motivation of volunteers with environmental assets in a community setting: the most important personal asset being the confidence to have conversations with people about a sensitive topic, such as alcohol.

**What is known about this topic and what this paper adds**

- Community‐centric approaches are increasingly encouraged to address public health issues.
- Reducing alcohol harm is a global and national key priority due to related health, social and economic costs.
- Health champion models are shown to have potential to improve the public's health.
- This is the first time a place‐based alcohol health champion (AHC) programme has been implemented.
- Volunteer AHCs were willing and confident to deliver alcohol advice, in contrast to attitudinal barriers commonly reported by healthcare professionals.
- AHCs’ views of intervening in alcohol licensing differed. Some felt comfortable sharing knowledge on how to refer issues to relevant authorities, others preferred a direct approach with managers of licensed premises.

## 1 INTRODUCTION

Globally, alcohol harm is recognised as one of the greatest risks to the population and impacts on the health‐related Sustainable Development Goals (World Health Organization, 2018). Reducing alcohol harm is a key priority for the UK Government due to the resulting harms to health, social and economic costs, including direct, indirect and intangible costs (Bhattacharya, 2017; Public Health England [PHE], 2016).

National and global policy encourages a community‐centric approach to empower individuals and groups to address collective needs locally (HM Government, 2010; Labonte & Laverack, 2008). Community engagement in public health interventions is known to enable people to have some control and empowerment over their own health (Brunton et al., 2017; O’Mara‐Eves et al., 2013). Using a health champion model has been shown to have potential to improve the public's health and to start changing cultural understandings of the health of communities (Woodall et al., 2013). Individuals volunteer to improve the health and wellbeing of their communities and families following training. This is done through outreach, communication of health messages, and signposting to relevant support services, using their skills and influence to motivate and empower their communities (PHE, 2015; National Institute for Health and Care Excellence, 2016). Through CICA, alcohol health champions were trained in providing alcohol identification and brief advice (IBA) to reduce alcohol harm; and influencing the availability of alcohol in their communities through intervening in alcohol licencing. Alcohol IBA refers to opportunistic identification of alcohol misuse and the delivery of brief advice; widely known as alcohol screening and brief interventions (Lavoie, 2010). Alcohol IBA describes a simple conversation aimed at those at risk of harm from their drinking, particularly people not experiencing any health problems. IBA has been shown to help at least one in eight drinkers reduce their alcohol intake (HM Government, 2012). IBA relies on the use of an ‘identification’ tool to identify a person's level of risk, followed by advice or onward referral to encourage behaviour change (Thom et al., 2016). AUDIT‐C was an identification tool used in CICA. It comprises three questions regarding the consumption of alcohol and can be adapted for easy use in the form of, for example, a scratch card. Scratch cards were given to all AHC teams as resources to use when working in the community.

The Communities in Charge of Alcohol (CICA) programme drew influence from asset‐based community development (ABCD) approaches by training community members across nine local government areas (‘local authorities’) in Greater Manchester to become Royal Society for Public Health (RSPH) Level 2 trained alcohol health champions (AHCs). AHCs were trained in informal alcohol identification and brief advice (IBA; or brief advice) to reduce alcohol harm and, to get involved in the alcohol licensing process. Brief advice conversations aimed to reach individuals drinking at hazardous levels as an early intervention to prevent harmful, higher‐risk drinking (Cook et al., 2018). CICA set out to strengthen existing community assets, such as people's values, capacity, skills, knowledge, connections and potential (Blickem et al., 2018; Foot & Hopkins, 2010; PHE, 2015; Rippon & Hopkins, 2015) and to build capacity in local communities. One of the roles of an AHC was to help train other community members to be AHCs to build sustainability of the progreamme within local areas. This was done through a ‘train the trainer’ approach where AHCs were given the skills to support future training sessions, for example, how to present information to others effectively and confidently.

Complex intervention implementation is a continually changing process (Craig et al., 2019; May, 2013). Barriers and facilitators to getting an intervention started are often overlooked in the reporting of interventions (Watson et al., 2018). Understanding the AHCs’ experiences of becoming involved in community approaches to licensing decision making and how they embedded knowledge and skills could inform intervention development and effective adoption elsewhere (O’Cathain et al., 2019). Therefore, this paper explores how newly trained AHCs perceive, experience and understand their training and role and how they start to embed it within their communities.

## 2 METHODS

The findings of this paper sit within the context of the wider evaluation of CICA (Cook et al., 2018; Ure et al., 2021) which explored the experiences of a range of stakeholders, including Licensing Officers, local CICA coordinators, and AHCs. A brief description of the role of an AHC is provided in Table 1. The AHCs became familiar with this role descriptor during their training. As per the protocol (Cook et al., 2018), training of AHCs took place between September 2017 to March 2019 in specific intervention areas across nine local authorities in Greater Manchester in the United Kingdom (UK). Each area was chosen based on small geographic areas affected by multiple health and social inequalities (Cook et al., 2018; Ure et al., 2021).

**TABLE 1:** Alcohol health champion (AHC) role descriptor (adapted from a plain English role descriptor used to recruit and train AHCs)

Alcohol Health Champion Role	Description
What does an Alcohol Health Champion (AHC) do?	Talks about the harms associated with alcohol and gives alcohol‐related brief advice to people. Helps communities have a say about alcohol availability in their community. Trains others to become AHCs using the ‘train the trainer' approach.
What AHCs receive	Two days’ training to gain knowledge and skills needed to improve community health and influence how alcohol is sold. Level 2 Royal Society for Public Health (RSPH) qualification^a^
Ways of using knowledge and skills gained in the training	Engage in informal conversations about alcohol and health with family, friends, and colleagues. Support people to reduce drinking through brief advice and/or guiding them towards specialist services. Attend local community social events to speak to people about alcohol and wider health issues. With support of other AHCs, local NHS services, the local authority or other organisations, attend events to promote a healthier relationship with alcohol. Provide support for communities to get involved with licensing decisions by helping them raise issues with the local authority about venues selling alcohol. Work with other members of the community and professionals to influence alcohol policy in local area and beyond.

^a^ In an English context, a level 2 qualification is at the same level as the General Certificate of Education (GCSE), an examination usually taken at age 16.

A mixed methods approach explored the views and understandings of the AHCs at baseline. First, all AHCs were invited to complete pre‐ and post‐training attitudinal questionnaires. The attitudinal survey was kept deliberately short (four questions) to limit the demand on participants given the intensity of the training provision. Participants also completed a self‐assessment of current alcohol use, using AUDIT‐C at their initial training event. Descriptive statistics, using IBM SPSS Statistics Version 26, were used to summarise AHC demographic characteristics and current level of drinking as categorised by their AUDIT‐C score. Related samples sign test statistics were conducted to ascertain changes in attitude following training. Post‐training, a purposive sample of AHCs (*n* = 5) were interviewed to explore early experiences of putting their new skills into practice (see Table 2 for AHCs’ motivations for taking part in CICA, which formed the criteria for meeting the purposive sampling aims). The following purposive selection criteria were met across the sample: (a) Family experiences of alcohol misuse/concerned relative; (b) Cares for and values the community/wants to help people; (c) Has lived experience of alcohol dependence/in recovery; (d) A general desire to learn about alcohol/increase alcohol awareness; (e) Works in the local community; and (f) Wants to gain a qualification. The purpose of the post training interviews was to explore how AHCs made sense of their role at this early stage, to give nuance and understanding to the questionnaire data, and thus a fuller view of the context in which the intervention was being established. Semi‐structured interviews were chosen as the most appropriate method of data collection, as they give voice to participants’ own perspectives and meaning (Braun & Clarke, 2013). Time since initial training was <3 months (*n* = 4) and 6 months (*n* = 1). Data collection comprised a mix of telephone (*n* = 2) and face‐to‐face (*n* = 3) interviews, ranging from 23 to 47 min in length. They were audio‐recorded, transcribed verbatim and anonymised. Face‐to‐face interviews took place in private spaces within community settings. AHCs from three CICA areas were interviewed. The relatively small sample size also reflects the limited pool of newly trained AHCs during the limited time available for the interviews, the timing over a holiday period, and facilitating interviews with AHCs who had pressing priorities outside the role.

**TABLE 2:** Characteristics of the study areas and participants at the time of the interviews

Participant/area	Characteristics
**Area 6**	**At the time of the interviews:** **CICA intervention co‐ordinated by the drug and alcohol service.** **One initial training session conducted, and no cascade training rolled out yet between the start of the intervention (September 2017) and the interview (July 2018).** **CICA training/knowledge/skills beginning to be implemented at wider community support events (e.g., coffee mornings).**
Peter, Area 6	Motivation to be an Alcohol Health Champion (AHC): in recovery from harmful drinking. Interview took place within 3 months of initial training. Aged 51–60 years; white British ethnicity; male; qualified to NVQ Level 4–5; non‐drinker.
Darren, Area 6	Motivation to be an AHC: wanting to help others and in recovery from harmful drinking. Interview took place within 3 months of initial training. Aged 51–60 years; white British ethnicity; male, qualified to NVQ Level 2/GCSE/O Level; non‐drinker.
**Area 8**	**At the time of the interviews:** **CICA intervention co‐ordinated by health and wellbeing service.** **One initial training session and two cascade training sessions had rolled out since the start of the intervention (September 2017) and the interview (July 2018).** **CICA training/knowledge/skills started to be implemented at community events (e.g., summer carnival).**
Amy, Area 8	Motivation to be an AHC: personal interest and desire to learn. Interview took place within 6 months of initial training. Aged 22–30, white British ethnicity; female; qualified to NVQ Level 3/A Level . At the time of the interview worked part time in a public house (pub; UK drinking establishment).
**Area 9**	**At the time of the interviews:** **CICA intervention co‐ordinated by health and wellbeing service.** **One initial training session and two cascade training sessions had rolled out since the start of the intervention (September 2017) and the interview (July 2018).** **CICA training/knowledge/skills beginning to be implemented at wider health promotion community events.**
Kathryn, Area 9	Motivation to be an AHC: third party harm, affected by alcohol dependency in family. Interview took place within 3 months of initial training. Aged 41–50 years, white British ethnicity; female
Grace, Area 9	Motivation to be an AHC: wanting to make a difference in community. Interview took place within 3 months of initial training. Aged 31–40 years, Black African ethnicity; female.

Abbreviations: A Level, advanced level (usually taken at age 18, equivalent to NVQ level 3); GCSE, General Certificate of Education (usually taken at age 16, equivalent to NVQ level 2); NVQ, National Vocational Qualification, NVQ level 4: equivalent of completion of the first year of a bachelor's degree; NVQ Level 5, equivalent of a foundation degree, attained after two years of completing a bachelor's degree; O Level, ordinary level (usually taken at age 16, replaced in 1988 by the GCSE).

A thematic analysis was conducted using a framework approach (Ritchie et al., 2014). A priori themes (e.g. from the interview guide/literature) were combined with themes identified from the interview transcripts. Framework analysis allowed a systematic approach to mapping and managing the data (Gale et al., 2013). See Table 3, for an overview of the analysis processes undertaken (Ritchie et al., 2014; Ure et al., 2021). The standards for reporting qualitative research (SRQR) were met during each stage of this study (O’Brien et al., 2014). Other steps to maintain the quality of the process included: verbatim transcription, checking transcripts against recordings, being reflexive and exploring data in a nuanced manner (Braun & Clarke, 2013, 2019).

**TABLE 3:** Summary of thematic analysis of the interviews using the framework approach

Stage of analysis	Processes undertaken
Familiarisation	All interview transcripts (*n* = 5) were read to re‐familiarise the researcher with the content. Initial notes and codes were generated at this stage (SCH)
Identifying a thematic framework	An initial framework was identified using a combination of the interview guide and the familiarisation codes (SCH). Initial framework was discussed with other researchers to sense‐check them (SCH/CU/MC)
Indexing	All transcripts were imported into QSR International NVivo 12 and coded systematically. NVivo was used to create a report of the quotes from the transcripts sorted into themes by interview participant (SCH)
Charting	A framework matrix was created in NVivo and then exported into Microsoft Excel. Columns represented themes and sub‐themes and the rows represented participants. This was to enable transparency of the data for reference during the interpretation process and for future analysis (SCH)
Mapping and interpretation	The framework matrix was used to synthesise and establish connections and associations across the themes, and between participants. Themes were continually refined during the write‐up of the results (SCH/PC/CU/MC)

Ethical approval was granted by the University of Salford research ethics committee in May 2017 (HSR1617‐135). Written consent was gained from participants prior to pre‐ and post‐training questionnaire completion. Separate written consent was provided at the time of interview following a period of consideration of a participant information sheet. A one‐off payment to cover travel and time costs was given to interview participants.

## 3 Findings

### 3.1 Questionnaires

In total, 93 out of 95 AHCs completed the pre‐ and post‐training questionnaires. Nearly three quarters (74.2%) of AHCs participating in the evaluation were aged between 31 and 60 years old. More women took part than men (61.3% female) and the majority of AHCs (69.9%) self‐identified as being of white ethnicity. Educational qualifications varied, with 9.7% reporting having no qualifications and nearly half (49.5%) having either GCSE/NVQ Level 2 (national qualifications typically taken at age 16), or A Level/NVQ Level 3 qualifications (typically taken at age 18) (Table 4). Almost two‐thirds (65.6%) who took part in the AUDIT‐C questionnaire either did not drink alcohol at all or were classed as lower‐risk drinkers (Table 4). Corroborating this, at the pre‐questionnaire stage, 89.2% of the participants agreed/strongly agreed with the statement that they ‘try to live a healthy lifestyle by not drinking too much’ (Table 4).

**TABLE 4:** Characteristics of AHC trainees who completed pre‐ and post‐training questionnaires

	AHC participants	
	*n*	%
Sex		
Male	36	38.7
Female	57	61.3
Age group		
18–21	2	2.2
22–30	12	12.9
31–40	16	17.2
41–50	29	31.2
51–60	24	25.8
61–65	3	3.2
65+	5	5.4
No answer given	2	2.2
Ethnicity		
White	65	69.9
Asian/Asian British	3	3.2
Black/African/Caribbean/Black British	4	4.3
No answer given	21	22.6
Highest qualification gained^a^		
No formal qualification	9	9.7
NVQ L2, GCSE, O Level or equivalent	25	26.9
NVQ L3, A Level, AS Level or equivalent	21	22.6
NVQ Level 4–5, Certificate of Higher Education or equivalent	3	3.2
NVQ L6, undergraduate degree or equivalent	17	18.3
Other	2	2.2
No answer given	16	17.2
Level of drinking (AUDIT‐C questions)		
1–4 lower risk drinking	61	65.6
5–7 increasing risk drinking	19	20.4
8–10 higher risk drinking	11	11.8
11–12 possible dependant drinking	1	1.1
Missing data	1	1.1
Participants “try to live a healthy lifestyle by not drinking too much”		
Agree/strongly agree	83	89.2
Neither agree nor disagree	5	5.4
Disagree/strongly disagree	4	4.3
No answer given	1	1.1
Total	93	100.0

^a^ GCSE = General Certificate of Secondary Education qualifications, with assessments usually taking place at aged 16 years. NVQ = National Vocational Qualification: a practical, work‐based award achieved through assessment and training. A Level = General Certificate of Education Advanced Level, with assessments usually taking place at aged 18 years.

Post‐training, nearly all AHCs agreed/strongly agreed that they felt more confident that they could talk about the harms associated with alcohol and give alcohol‐related brief advice than they did pre‐training (91.4% compared with 79.6%) (*p* < 0.001). Post‐training, the number of AHCs who reported community engagement in alcohol availability as important increased from 91.4%, to 92.5% (*p* = 0.001). Furthermore, AHCs felt more confident post‐training that they could raise issues about venues selling alcohol (90.3%, compared with 74.2% pre‐training) (*p* < 0.001) (Table 5).

**TABLE 5:** Changes in attitudes towards conducting AHC activities following training

Positive differences	Negative differences	Number of ties	*N*	Related samples sign test statistic	*p* value	Pre‐training—agree/strongly agree with statement	Post‐training—agree/strongly agree with statement
Q1 I feel that it is important to promote healthy lifestyles and behaviours within my community							
11	7	71	89	11.0 (standardised test statistic 0.7)	0.480	96.8% (*n* = 90)	91.4% (*n* = 85)
Q2 I feel confident that I could talk about the harms associated with alcohol and give alcohol‐related brief advice to people							
33	9	46	88	33.0 (standardised test statistic 3.5)	*p* < 0.001*	79.6% (*n* = 74)	91.4% (*n* = 85)
Q3 I feel that it is important for communities to have a say in alcohol availability in their community and get involved in licensing decisions							
27	7	55	89	27.0 (standardised test statistic 3.3)	*p* = 0.001*	91.4% (*n* = 85)	92.5% (*n* = 86)
Q4 I feel confident that I could raise issues about venues selling alcohol							
43	9	37	89	43.0 (standardised test statistic 4.6)	*p* < 0.001*	74.2% (*n* = 69)	90.3% (*n* = 84)

* Significant.

### 3.2 Interviews

To provide context, Table 1 details characteristics of interview participants and the type of service provider, from here on referred to as local CICA co‐ordinator, supporting their involvement in CICA. Motivations to take part in CICA ranged from own personal struggles with alcohol use (‘in recovery’) or family experience, to a wish to learn more and do volunteering in the community. Each participant and area have been anonymised. Area codes are the same as used in other publications on CICA. Areas differed in the extent that cascade training had been rolled out (see sister paper for more findings on intervention roll out (Ure et al.,  2021)).

**FIGURE 1:**
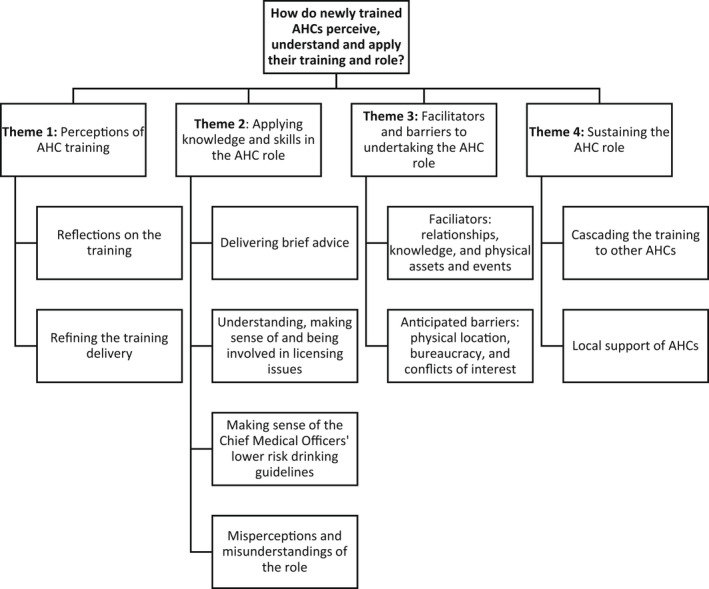
Thematic map: early perceptions and experiences of AHC role

Four overarching themes were identified (Figure 1): (a) perceptions of AHC training; (b) applying knowledge and skills in the AHC role; (c) barriers and facilitators to undertaking the AHC role; and (d) sustaining the AHC role, including thoughts on cascading the training to others.

### 3.3 Theme 1: Perceptions of AHC training

#### 3.3.1 Reflections on the training and refining training delivery

Participants expressed pleasure from the training and gaining new knowledge, reporting that they *‘enjoyed it’* and ‘*learned quite a lot’* and that it was a positive experience knowing that they *‘can help people’*. However, the training content was considered by some AHCs as a lot to fit into a short space of time. Changes in both the language and structure of the training were suggested as something that would be beneficial:

That was difficult for me that day…and [the trainer] was rushed…I would stagger it and I would change some of the language away from what was delivered…I mean it’s not like a job where you’ve got the time to do the induction and ask a colleague and get to know the language. (Peter, Area 6)

…I thought it was a bit too much in a short period of time. If we could sort of spread it, then maybe…we had a lot of handouts. (Grace, Area 9 **)**

The intensity of the course may not have left enough time for AHCs to explore and evaluate information or to check that all participants had properly understood. There were occasions where misperceptions arose. This included Kathryn, when recounting her experience of an off‐licence shop selling alcohol to her intoxicated relative:

…That rule I think should be put on shops as much as pubs so that a law should be made that if they come in in an absolute state [drunk] you should not be allowed to sell them that alcohol…but they can’t if the law isn’t there. (Kathryn, Area 9)

However, the Licensing Act 2003 prevents the sale of alcohol to a person who is drunk in both on‐ and off‐licensed premises, which had been iterated in the training session.

### 3.4 Theme 2: Applying knowledge and skills in the AHC role

#### 3.4.1 Delivering brief advice

Although early in their role, all participants mentioned that they had experience of giving alcohol advice, and they mentioned a range of settings where this had taken place. This ranged from formal, including attending events where their attendance had been facilitated by their coordinator, coffee shops, and informal, for example conversations with friends and families. Each of the participants spoke about their experiences of giving brief advice about alcohol and signposting to other appropriate services if more specialist support was needed. AHCs delivered information in various ways, for example, in everyday conversation, or as part of wider health and wellbeing advice. All had used brief advice tools, including the AUDIT‐C scratch card or an ‘alcohol wheel’ to demonstrate the number of alcohol units and calories in each drink. An alcohol unit is a measure used in the UK of how much alcohol is in a drink. One unit is 10ml or 8g of pure alcohol; the amount of alcohol the average adult can process in an hour (NHS, 2018). Some AHCs reflected on the importance of offering non‐judgemental advice and to approach the subject gently, giving the conversation a ‘*natural flow*’ because they did not want to make people ‘*feel uncomfortable*’. Brief advice on swapping and reducing alcoholic drinks was friendly rather than authoritarian:

…I don't kind of preach it to them…I would alternate an alcohol drink with a soft drink to try and reduce the amount of units that they're taking in. (Darren, Area 6)

#### 3.4.2 Understanding, making sense of, and being involved in licensing issues within the AHC role

The AHCs demonstrated their knowledge of the Licensing Act 2003 from their training. Most were aware of the requirements for responsible retailing regarding drinks promotions and checking the age and level of intoxication of individuals purchasing alcohol in accordance with the legislation. Peter, for example, noted that a supermarket in his local community had prominent displays of alcohol in the entrance, which, although not strictly regarded as irresponsible retailing in the Act, was considered by him as drawing attention to the alcohol:

…the first thing you get as you walk through the doors is there’s literally a stand out in the front of the door and the first thing they’ve got on there was alcohol. (Peter, Area 6)

However, a different supermarket was reported as showing responsibility by being strict on under‐age alcohol sales and not selling alcohol to those perceived as intoxicated:

…the girls asked for identification [from underage customer] …the girl who was service on the till refused to serve one gent ‘cos she actually said ‘in my opinion you’ve had enough to drink already’…it just shows that some stores are taking a stance. (Peter, Area 6)

While some AHCs reported a preference for direct action, for instance by approaching managers of licensed premises, others preferred educating other community members in how to tackle licensing problems. The AHCs highlighted how the training had increased their awareness of the powers available to communities and the need to work collaboratively to address licensing issues:

… if it’s in a residential area, if it’s close to housing, I would say ‘have you spoke to your neighbours?’…and if you get together as a group and then approach as a group, and I would also say to them don’t feel that you can’t actually get your local council involved. (Darren, Area 6)

…And it’s knowing that I can talk if somebody came to me complaining or even just an informal discussion with them about their experience with their neighbourhoods then I could encourage them or empower them to go and challenge whatever’s happening. (Grace, Area 9)

#### 3.4.3 Making sense of the UK Chief Medical Officers’ lower risk drinking guidelines

AHCs demonstrated a nuanced understanding of the UK lower risk drinking guidance (up to 14 units of alcohol per week; one unit = 10 ml or 8 g of alcohol). AHCs felt that they are *‘just a recommendation’* and *‘a guide’* and that within those guidelines, people should still be careful. The AHCs showed understanding that people's responses to alcohol consumption varied, which they shared when giving brief advice, as expressed by Darren:

….it’s a guide ‘cos what you will find, you can have two people of the same body mass, and one person will get drunker quicker than the other person…you just need to be aware that not everybody’s body is the same… (Darren, Area 6)

Amy felt that advice needed to be individualised and even people who were drinking less than the recommended upper limit of the guidelines might still value guidance on reducing alcohol intake:

…I’d still encourage them to cut it down…I’d just go more along the line of well do you think that’s too much? What do you want to be drinking a week? Yeah I think they’re [the guidelines] good just for people to get an idea of where it is that could affect your health massively. (Amy, Area 8)

#### 3.4.4 Misperceptions and misunderstandings about the role

The intended role of an AHC was to reduce alcohol‐related harm by intervening early through either individual or community action. Informal conversations using the principles of IBA was aimed primarily at reaching those drinking at hazardous levels to prevent harmful, higher‐risk drinking. However, some AHCs viewed CICA as largely directed at those already drinking harmfully, who may possibly be alcohol dependent:

I think [different area] would be better to hit on. In [place]…I’ve seen a lot more alcoholics up there than anything that touches a little bit of this place. (Kathryn, Area 9)

The principle of CICA was to have informal conversations where appropriate, with a focus on the quality of the conversations with people who may not otherwise have accessed alcohol advice. There were no pre‐set targets for numbers of conversations to be carried out by AHCs. However, some participants were concerned about not having enough conversations with community members around alcohol:

Everyone feels like they're bring…they're not getting enough numbers. (Peter, Area 6)

### 3.5 Theme 3: Facilitators and barriers to undertaking the AHC role

#### 3.5.1 Facilitators: relationships, knowledge, physical assets and events

Facilitators to the role were identified as: positive relationships between the AHCs enabling them to support one another in their roles; personal attributes and knowledge already possessed by AHCs and key community members; and the availability of local physical assets such as libraries and community centres that could be utilised for having conversations. For example, Darren already had an established volunteer role in his local library (a key physical asset) coordinating a weekly coffee morning. This gave him the opportunity to network with key, community‐based individuals (such as the library manager) and put the CICA training skills into practice, where appropriate, at the coffee mornings with local community members. This started almost immediately after attending the initial training:

…I run an informal coffee morning where there’s basically no boundaries to what we can talk about….and the new manager who’s coming in, she’s all about the library being used as a community hub. (Darren, Area 6)

Other existing and established community events were facilitators to the AHC role. Summer carnivals and attendance at health and wellbeing events, such as wider health promotion events (e.g. incorporating blood pressure tests) enabled AHCs to deliver alcohol advice as part of a larger volunteer team. At these community events, AHCs saw advantages of having a presence alongside drug and alcohol service providers. The informal nature provided a degree of anonymity for those seeking advice without the commitment of seeking out formal alcohol services:

…at the carnival and the stalls…I think it’s so light‐hearted…and they are just willing to chat…a couple went to the back of the stall and actually spoke to [local coordinator] about serious things that they’re facing…that’s like just off the carnival. (Amy, Area 8)

Grace described how AHCs in Area 9 worked alongside other volunteers and healthcare providers as part of wider health promotion events outside the scope of CICA, using the opportunity to offer brief advice about alcohol use.:

As an alcohol health champion, I have taken people’s blood pressure and advised them on how they can cut down on their drinking…signposted them to their GPs…yeah, I did that as part of a community. (Grace, Area 9)

Life experiences of AHCs were reported as key to the role and these included being an active member of a community and being a community‐minded individual:

Over the years I’ve just blended in with the community and done things…it's about connecting on a community level. (Peter, Area 6)

#### 3.5.2 Barriers: physical location, bureaucracy and conflicts of interest

In this early phase of the intervention AHCs had not experienced many barriers in offering brief advice, although they were aware of potential barriers. One example was in setting up a drop‐in service, which was felt to *‘restrict people’* who were concerned they might be seen attending a particular location, such as a specific café, that they would not normally attend. There were also concerns that members of the wider community might be *‘a bit aggressive’* towards AHCs, although these were hypothetical concerns at this stage of their role.

Negotiating licensing processes was viewed as a potential barrier, *‘full of bureaucracies’*. However, when faced with this, Peter felt that it was *‘easier to go in’* to the licensed premises to discuss issues directly than go through the paperwork of putting in an official representation (commenting/complaining in writing to the licensing authority), thus working around the barrier showing a positive example of AHC action. Peter applied his new knowledge of alcohol licensing legislation (Licensing Act 2003) to talk to an initially defensive manager of a pub (public house; licensed to sell/supply alcohol ‘on the premises’), who felt that issues of litter, empty glasses and noise around his premises were *‘not a licensing issue’*. To maintain a positive relationship with the local community, Peter reminded the manager of his responsibility to the neighbourhood and this had the positive outcome of reducing some of the anti‐social behaviour around the premises:

Clear the glasses, tell them if they want a drink go inside but don’t have them sitting out at two and three in the morning chatting away because you’re making money…He said yeah but when I shut the door they’re outside they can do what they want. Well then you need to know, if they’re your clients at the pub, that there’s kids across the road that need sleeping…That’s how it went…’cos he was being all defensive like, it’s outside the pub, it’s not a licensing thing…and we’re neighbours. (Peter, Area 6)

An example of a more definite barrier was identified by Amy, who had a potential conflict of interest regarding taking licensing action due to working part time in a pub. Amy felt ambivalent about raising concerns about a pub setting:

Yeah, I’m not a complainer really. That’s just me…I probably wouldn’t do it for a pub, but I might do it for a shop. ‘Cos I think pubs have to earn a living and I don’t know. I think if you’re going into a pub, you know you want a drink. If you’re going into a shop, I don’t know… (Amy, Area 8)

Perhaps more pervading was a socio‐cultural barrier to making complaints within a community: *‘We don't grass each other up’* (Peter, Area 6).

### 3.6 Theme 4: Sustaining the AHC role

#### 3.6.1 Cascading the training to others

At the time of the interviews, four AHCs were looking forward to cascading the training to new AHCs and one had already been involved in a cascade training session. They suggested ways in which the original training could be developed. There was a feeling that the training needed *‘to be slower’*, perhaps held over more days, and the content needed simplifying to ‘*change the language*’. They felt that they required a refresh of the information and some time to ‘*carry out some more research about it*’ before they cascaded to others. Overall, with some of those changes in place, AHCs were confident in and positive about planning the cascade training, not just in their own communities, but also beyond the study intervention area:

Well hopefully, like I’ve said to [local coordinator], we can then, once we’ve done a bit of training, we can maybe see about extending it to other areas…we can widen the net out. (Darren, Area 6)

However, there was a feeling that their role in cascade training in the future should be in assisting, rather than leading the training sessions, which they felt less confident about:

I loved it, it was brill…when somebody is on their own…at least you can partner up with them and they don’t feel on their own, having to struggle…. I would love to keep helping…but I don’t fancy doing it on my own…If I had to teach one person I could do it. If I had to teach a whole class, no. (Katherine, Area 9)

The CICA training programme was accredited by a professional body, the RSPH, and this was generally seen as positive when recruiting new AHCs because it cemented its importance and identified it as ‘*a properly structured piece of training’* that gave the programme a level of approval. Some felt that the RSPH accreditation and qualification was *‘important’* but that there should be some other incentive offered alongside that *‘might be more meaningful’* to new trainees:

…tie it in with something that they need…you can get points and credit towards some education…a bus pass or something…a recognition. (Peter, Area 6)

However, the use of accredited training also led to delays because, in some areas, the training centres first needed to be accredited by RSPH. This was frustrating to some AHCs and contributed to difficulties in keeping momentum and retaining new recruits:

We’ve got a number of people interested…in doing the course…it’s this delay of getting the accreditation to do it, it’s the longer it’s going on it might, they might just think ‘oh you mentioned this two months ago’…it’s when you keep putting it off, that’s when you wonder whether or not they’ll just think ‘oh I can’t be bothered now’. (Darren, Area 6)

#### 3.6.2 Local support of AHCs

Notwithstanding the types of activity AHCs explored in their role, there was a clear reliance on the local CICA coordinator. For some, having a lead professional located within a local health and wellbeing service appeared to offer a sense of validity:

‘It's not as though we're trying to do it as a community thing without any backing, we're actually doing it with the backing behind us’. (Darren, Area 6)

For others, the personal attributes of their local coordinator made a significant impact on their own sense of self‐efficacy and that without the coordinator's encouragement they would not have stayed involved.

Knowing that the local CICA coordinator was available further supported the AHCs’ confidence in the role. This seemed to be particularly helpful in enabling AHCs to manage their role boundaries effectively during brief advice conversations. If the level of information disclosed needed further signposting and support, having quick access to their coordinator by email or phone provided reassurance that the AHC was not alone.

## 4 DISCUSSION

To the best of our knowledge, CICA is the first community‐led, place‐based alcohol health champion (AHC) programme to be implemented. A significant finding was that AHCs were confident in providing alcohol advice to family members, friends and strangers in their communities. These findings are a stark contrast to the significant barriers commonly reported by health and community‐based professionals when implementing alcohol identification and brief advice (Derges et al., 2017).

Within the first 3 months of their new role, AHCs broadly had confidence that they could help community members raise concerns about harms related to alcohol licensing decisions. Questionnaire data showed a significant increase in confidence post‐training, while qualitative interview data indicated preferences for both informal or formal mechanisms to influence the alcohol environment. Personal experiences appeared to influence the extent to which an AHC may get involved in the licensing aspect of the role: impacted by conflicts of interest; lack of optimism; or fear of repercussions if seen to be an informant. However, CICA demonstrates the active role that AHCs were willing to adopt in licensing, adding new insights into community engagement in licensing decision‐making beyond a ‘story‐telling’ role (Reynolds et al., 2020). These findings are important, since the philosophy behind an assets‐based approach is that volunteers have a unique ability to connect to members of their own community, understand their own circumstances and have reasons to improve the health of their community (Lindstrom & Eriksson, 2005).

The importance of ensuring that role boundaries are clear when recruiting and training volunteers to help them to undertake a role, with active management of issues through accessible support and ‘light touch’ supervision from professionals (South et al., 2012). Being able to access ongoing support and supervision through their local CICA coordinator was valued by AHCs, thus providing volunteers with opportunities to continue to learn and correct misperceptions. For example, those who held the view that the intervention should concentrate on higher risk, dependent drinkers most visibly experiencing alcohol harm could stereotype those ‘in need’ as being those with only the most severe drinking patterns. This could lead to missed opportunities for early identification of at‐risk individuals; a potential unintended negative consequence of CICA identified a priori in CICA’s ‘dark logic’ model (Cook et al., 2018).

While barriers to the role were anticipated, at this stage of the intervention they appeared to be hypothetical among those interviewed, and a range of facilitators were identified. These facilitators included physical assets that provided opportunities to carry out the AHC role, such as access to community buildings and wider community events. As identified, relationships with the local CICA co‐ordinator and other AHCs helped to develop solidarity within the experience as well as the self‐belief to exercise their role after the training. AHCs brought with them a willingness and commitment to make early progress.

Previous evaluations of other (generic) health champion roles have taken a view across the whole period of an intervention (Woodall et al., 2013) and reflected on how relationships between champions and their role develop over time (Van Laere & Aggestam, 2016). The early implementation of an intervention with multiple components is a changing process involving other influences (May, 2013). Such influences include, for example, the existing infrastructure in place at the start of the intervention (Ure et al., 2021); the external agency support from those coordinating the interventions in each area; and wider support of the programme. This early look at how AHCs were mobilising and establishing their role give an important baseline with which to compare experiences after having been in the role for a longer time (to be reported in due course).

### 4.1 Limitations and strengths of the study

There were limitations to this phase of the evaluation, in particular the small sample size for the interviews. Whilst they gave an in‐depth insight into the knowledge, understandings and experiences of the AHCs, it would have been advantageous to have voices representative of a wider variety of intervention areas including AHCs who may have become inactive, and we acknowledge that this may have led to a degree of selection bias. It was not possible to approach AHCs who had become inactive since, by definition, they were not in touch with their coordinators. The pool of potential participants was limited by the time frame of the interviewing component of this study and the criteria that participants should be newly trained, given that training had been spread out over 18 months, but the interviewing component took place over a month. Despite there not being any set targets of number of brief interventions for each area to reach, asking AHCs about their activity may have also increased Hawthorne effects (Audrey et al., 2019), creating a feeling among participants that they were not doing ‘enough’. Nevertheless, they provide an in‐depth view of how AHCs made sense of, adopted and valued the new responsibilities and tasks involved in their role at this early stage (May et al., 2015). The profile of participants in CICA demonstrated that the findings are likely to be transferrable to other community contexts with high levels of deprivation when implementing similar AHC programmes.

## 5 CONCLUSIONS

A 2‐day training course for volunteer AHCs increased feelings of confidence to get involved both in alcohol licensing at a community level and having informal conversations to promote healthier relationships with alcohol at an individual level. The CICA intervention focused on specific small communities that were affected by multiple health and social inequalities, making finding and recruiting motivated volunteers challenging, as described for CICA elsewhere (Ure et al., 2021). However, these findings suggest that volunteers and communities have significant strengths to bring to the role. Perhaps the most important of these is the confidence to have conversations with community members about a sensitive topic such as alcohol.

## CONFLICT OF INTEREST

KA is a trustee of the RSPH. SA is a member of the NIHR public health research board. All other authors declare that they have no competing interests.

## AUTHOR CONTRIBUTION

PAC is the Principal Investigator. PAC and KA made substantial contributions to the overall study conception. PAC and SA made substantial contributions to the overall study design. MC contributed to the analysis and advised on drafts of the manuscript. CU and EJB made substantial contributions to analysis of data and drafts of the manuscript. SCH led on data collection, analysis, synthesis and writing of the manuscript at all stages. All authors contributed to the writing and editing of the manuscript for publication and read and approved the final manuscript.

## Data Availability

The data that support the findings of this study are available from the corresponding author upon reasonable request.
